# Biallelic mutations in mitochondrial tryptophanyl‐tRNA synthetase cause Levodopa‐responsive infantile‐onset Parkinsonism

**DOI:** 10.1111/cge.13172

**Published:** 2018-02-05

**Authors:** E.A. Burke, S.J. Frucht, K. Thompson, L.A. Wolfe, T. Yokoyama, M. Bertoni, Y. Huang, M. Sincan, D.R. Adams, R.W. Taylor, W.A. Gahl, C. Toro, M.C.V. Malicdan

**Affiliations:** ^1^ NIH Undiagnosed Diseases Program, Common Fund, Office of the Director NHGRI, NIH Bethesda Maryland; ^2^ Movement Disorders Division School of Medicine, New York University Langone New York New York; ^3^ Wellcome Centre for Mitochondrial Research Institute of Neuroscience, The Medical School, Newcastle University Newcastle upon Tyne UK; ^4^ Office of the Clinical Director NHGRI, NIH Bethesda Maryland; ^5^ Section of Human Biochemical Genetics, Medical Genetics Branch NHGRI, NIH Bethesda Maryland

**Keywords:** medical genetics, Parkinsonism, precision medicine, tRNA synthetase

## Abstract

Mitochondrial aminoacyl‐tRNA synthetases (mtARSs) are essential, ubiquitously expressed enzymes that covalently attach amino acids to their corresponding tRNA molecules during translation of mitochondrial genes. Deleterious variants in the mtARS genes cause a diverse array of phenotypes, many of which involve the nervous system. Moreover, distinct mutations in mtARSs often cause different clinical manifestations. Recently, the gene encoding mitochondrial tryptophanyl tRNA synthetase (WARS2) was reported to cause 2 different neurological phenotypes, a form of autosomal recessive intellectual disability and a syndrome of severe infantile‐onset leukoencephalopathy. Here, we report the case of a 17‐year‐old boy with compound heterozygous mutations in *WARS2* (p.Trp13Gly, p.Ser228Trp) who presented with infantile‐onset, Levodopa‐responsive Parkinsonism at the age of 2 years. Analysis of patient‐derived dermal fibroblasts revealed decreased steady‐state WARS2 protein and normal OXPHOS content. Muscle mitochondrial studies suggested mitochondrial proliferation without obvious respiratory chain deficiencies at the age of 9 years. This case expands the phenotypic spectrum of WARS2 deficiency and emphasizes the importance of mitochondrial protein synthesis in the pathogenesis of Parkinsonism.

## 1 INTRODUCTION

Levodopa‐responsive Parkinsonism is the clinical hallmark of Parkinson's disease (PD) (MIM 168600), the second most common neurodegenerative disorder after Alzheimer's disease. While most patients with PD develop symptoms after the age of 50, a small subset of patients (5%‐10%) manifest early‐onset PD (EOPD)^1^, ^2^, ^3^ and are more likely to harbor recognized genetic risk factors.^1^, ^2^, ^4^ Levodopa‐responsive Parkinsonism presenting in infancy or childhood is extraordinarily rare and may occur as a comorbidity to other diseases or genetic conditions.^5^ From a neuropathology perspective, prototypical sporadic PD is regarded as a α‐synucleopathy pathologically associated with Lewy bodies. Other conditions emerging from progressive nigrostriatal degeneration that manifest clinical features of PD but lack Lewy bodies are referred to as Parkinsonian syndromes or Parkinsonism.

Accumulating evidence indicates that the pathophysiology of Parkinsonism involves mitochondrial dysfunction. For example, mitochondrial toxins are known to induce Parkinsonism in human and animal models and several genes directly linked to mitochondrial function and mitophagy, including DJ‐1 and PINK1, have strong associations with EOPD.^1^, ^2^, ^3^ Although, the mitochondrial aminoacyl‐tRNA synthetases (mtARSs) are key components of the mitochondrial translation machinery and are associated with disturbances of mitochondria function, they have not yet been associated with PD or Parkinsonian syndromes. Mutations in mtARS genes have been shown to cause a multitude of phenotypes, predominantly affecting the nervous system,^6^, ^7^ which can vary significantly depending on the specific mutations in a given mtARS. Recently, mitochondrial tryptophanyl‐tRNA synthetase, encoded by *WARS2*, has been associated with the phenotypes of intellectual disability, leukoencephalopathy and seizures.^8^, ^9^ Here, we describe a patient with pathogenic *WARS2* mutations presenting with infantile‐onset, Levodopa‐responsive Parkinsonism.

## 2 MATERIALS AND METHODS

### 2.1 Clinical data and samples

The patient, following informed and written consent, under the protocol 76‐HG‐0238, “Diagnosis and Treatment of Patients with Inborn Errors of Metabolism or Other Genetic Disorders” of the NIH‐UDP. Clinical data and patient samples were obtained in accordance with the ethical standards approved by the National Human Genome Research Institute (NHGRI) Institutional Review Board (IRB).

### 2.2 Genetic analysis

High density whole genome SNP array methods were done as described in Reference 10.

Whole exome sequencing of both parents and the patient was performed at NIH Intramural Sequencing Center (NISC). SureSelectV5 (Agilent Technologies) was used for exome capture and subsequent sequencing was done on a HiSeq2000 instrument (Illumina Inc.). Sample library preparation, sequencing, and analysis were performed using the standard NISC pipeline^11^ and Axeq Technologies (Seoul, South Korea). The analysis of whole exome sequencing data is described below.

Whole‐exome sequence analysis was performed on genomic DNA that was isolated from the patient and his unaffected mother and father. The quality of whole exome data is shown in Table S1 (Supporting information).

The sequencing reads then were filtered for quality, and aligned to human reference genome NCBI build 37 (hg19) using in‐house developed pipelines, one based on Novoalign (Novocraft Technologies), and separately a diploid aligner^12^ run on a commercial platform (Appistry Inc.). Variants were called with HaplotypeCaller and GenotypeGVCFs.^12^, ^13^, ^14^ Annotations utilized snpEff^15^ and a combination of publically available data sources (ExAC, GnomAD, ESP, 1000Genomes) and internal cohort statistics were utilized for variant filtration. We assessed the variants listed in the Variant Call Files (VCFs), which were filtered based on rarity (MAF <0.02, 95% confidence interval and homozygote count ≤25; UDP founders cohort population frequency data with variant allele count <8),^10^ Mendelian segregation, and predicted deleteriousness, and prioritized based on coding effect (non‐synonymous, frameshift, stopgain, stoploss, startloss, inframe), proximity to splice sites (within 20 base pairs of a canonical splice site into the intron, or 5 base pairs into the exon), CADD v1.3 Phred scores^16^ and Exomiser.^17^ The quality of alignment and genotype call of variants were checked using the Integrative Genome Viewer (https://www.broadinstitute.org/igv/home). The final candidate variants were validated through Sanger sequencing. The primers GCAACGTCACTACGCTCTGA (fwd) and GGGATCCAGGGAAAAACACT (rev) were used to amplify the region of genomic DNA around the c.37T>G variant and the primers TCTCAATCACAGCATCTGCC (fwd) and AAAACAGAATGGTTGTGTGGG (rev) were used to amplify the region surrounding the c.683C>G variant in *WARS2* (NM_015836.3). Sanger dideoxy sequencing of the PCR products was executed by Macrogen. The resulting sequences were aligned using Sequencher v.5.0.1 (Gene Codes).

Variant pathogenicity was evaluated by SIFT (http://sift.jcvi.org), PolyPhen (http://genetics.bwh.harvard.edu/pph2/), MutationTaster (http://www.mutationtaster.org), CADD Phred, and wInterVar (http://wintervar.wglab.org).

### 2.3 Analysis of dermal fibroblasts

A culture of dermal fibroblasts from the patient was established as previously described.^18^ Control fibroblasts were purchased from Coriell Institute for Medical Research. For quantification of WARS2 and oxidative phosphorylation in dermal fibroblasts, human fibroblasts were trypsinized, pelleted, and resuspended in cell lysis buffer as previously described,^19^ using the following antibodies: COXI (Abcam cat# ab14705), SDHA (Abcam cat# ab14715), UQCRC2 (Abcam cat# ab14745), NDUFB8 (Abcam cat# ab110242), MT‐ATP6 (ProteinTech cat# 55313‐1‐AP), WARS2 (kind gift from Prof. Roger D. Cox, and from SAB1406979, Sigma Aldrich) and α‐tubulin (Abcam cat# ab7291), and HRP‐conjugated secondary antibodies (Dako Cytomation).

### 2.4 Analysis of skeletal muscle biopsy

A piece of muscle was surgically obtained for enzymology studies (flash‐frozen), histology (OCT‐embedded), and electron microscopy (fixed in glutaraldehyde solution). Coenzyme Q10 (CoQ10) content was measured by high performance liquid chromatography with CoQ9 as an internal standard, and individual enzyme activities of the mitochondrial respiratory chain complex enzymes were measured by a diagnostic laboratory (Robert Guthrie Biochemical Genetics Laboratory, Buffalo, NY). Mitochondrial and nuclear DNA copy numbers were performed by a clinical lab using real‐time quantitative PCR (qPCR) specific primers for the mitochondrial tRNA^Leu(UUR)^ (*MT‐TL1*) gene and the nuclear β‐2‐micoglobulin (*β2M*) gene (Baylor Medical Genetics Laboratory).

## 3 RESULTS

### 3.1 Clinical description

The patient was the first and only child born to non‐consanguineous parents of European descent with no relevant family history. He was born at 37 weeks of gestation as a product of in vitro fertilization that was necessary because of tubal factor infertility. Due to premature labor that started at 23 weeks gestation and maternal bleeding related to placenta previa, he was delivered via cesarean section. Apgar scores were 9 and 9 out of 10 (at 1 and 5 minutes), with birth weight of 3302 g, length of 50.8 cm, and unknown occipital frontal circumference or OFC. Following delivery, he was noted to have pneumothorax and received a chest tube and was monitored on a ventilator for 4 days. He also developed a mild form of jaundice, but did not require phototherapy and was discharged at 5 days.

Early development was normal until the age of 1 when he was first noted to have left leg tremor while learning to stand. At 18 months, the left leg tremor spread to his right leg and he was noted to have expressive language delay despite good receptive language skills. By 23 months, tremor had also become apparent in the upper extremities along with intermittent dystonic posturing of all extremities (Videos [Link], [Link], [Link], [Link], [Link], [Link], [Link] in Appendix S1, Supporting information). Symptoms were severe enough to warrant a trial of Levodopa. After a trial dose of Levodopa, lower extremities tremor and dystonia disappeared for an entire day. A spinal tap revealed tetrahydrobiopterin (BH4), 5HIAA, and HVA levels that were in the low range of normal. Targeted testing for GCH1, phenylalanine loading test, and repeat CSF neurotransmitter study ruled‐out GTP cyclohydrolase 1‐deficient dopa‐responsive dystonia (Segawa's disease). A regiment of regular Levodopa therapy was instituted resulting in a stable period of 3 to 5 years with normal progression in acquisition of motor, language and social milestones. After another 2 years of gradual increases and adjustments in timing and dosing of Levodopa, the patient began to experience typical motor complications of more advanced PD including peak‐dose and wearing‐off phenomena with disabling retrocollic dystonic spasms.

**Figure 1:**
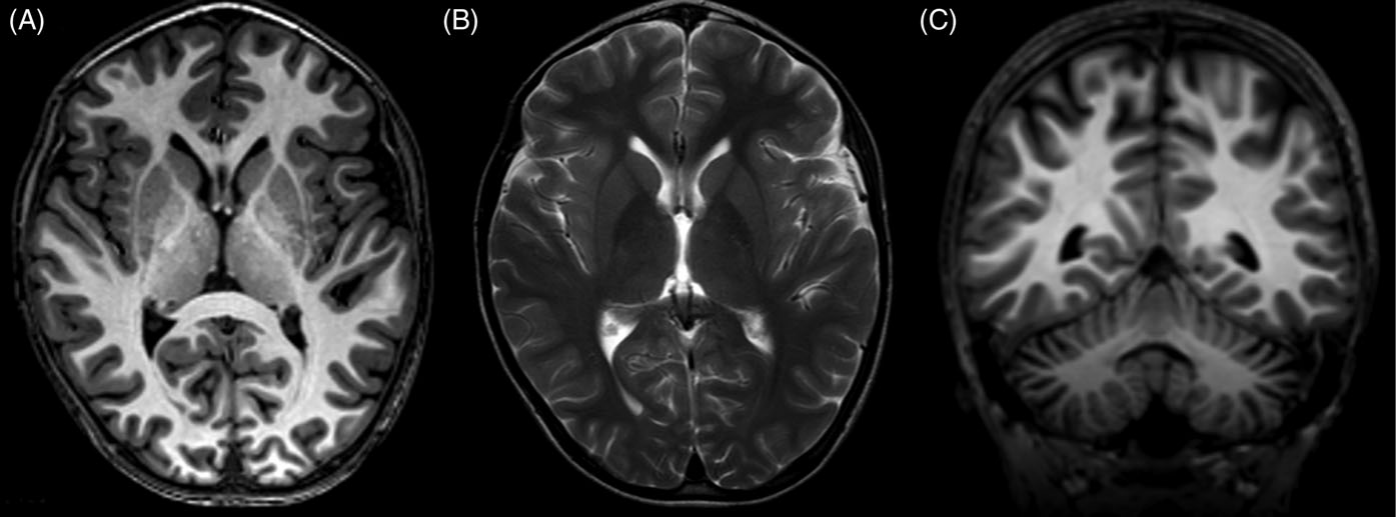
Clinical data: MRI images collected at the age of 9 years. MPRAGE (A) and T2‐weighted (B) axial images at the level of the basal ganglia and MPRAGE coronal images at the level of the middle cerebellar peduncles (C). Images were collected on a 3.00 T Achieva Philips Medical MRI scanner

The patient was evaluated at the NIH at age 9 and had advanced Levodopa‐responsive Parkinsonism with prominent and unpredictable “on‐off” fluctuations, peak‐dose dyskinesia, stooped posture and disabling off‐dystonia. Chronic headaches occurred due to retrocollic dystonic spasms. A brain magnetic resonance imaging (MRI) suggested progressive generalized brain atrophy, but there was no dysmyelination, leukoencephalopathy, or abnormalities of the basal ganglia (Figure 1A‐C). Qualitative evaluation of MRI spectroscopy revealed slightly low *N*‐acetylaspartate (NAA) in the left frontal white matter compared to normal adults (a reference spectrum from a normal age matched control is not available); the spectrum from this area appeared to be normal on the previous exam. In the left centrum semiovale, NAA appears to be slightly low compared to a normal age matched control. In the midline parietal gray matter, left basal ganglia, left thalamus, and superior cerebellar vermis, metabolite levels are unremarkable. The CSF voxel does not show significantly elevated lactate.

At age 10, he underwent implantation of deep brain stimulator (DBS) leads that resulted in substantial improvement in the motor complications of PD.

### 3.2 Clinical data and samples

The patient, following informed and written consent, was admitted in the NIH Clinical Center under the protocol 76‐HG‐0238, “Diagnosis and Treatment of Patients with Inborn Errors of Metabolism or Other Genetic Disorders” of the NIH‐UDP; clinical data and patient samples were obtained in accordance with the ethical standards approved by the National Human Genome Research Institute (NHGRI) Institutional Review Board (IRB).

### 3.3 Genetic analysis

Pathogenic SNPs, small insertions/deletions, and copy number and structural variants in the PD‐associated genes *PINK1*, *PRKN*, *PARK7*, and *SCNA* were ruled out by whole exome sequencing and whole genome single nucleotide polymorphism (SNP) array analysis. Top candidate variants were identified in several genes including *KDM5C*, *LRCH2*, *FRY*, *CUL4A*, *SETD6*, and *WARS2* (Table S2 in Appendix S1). Specifically, the patient was compound heterozygous for 2 variants in *WARS2* (NM_015836.3): a missense variant c.37T>G (p.Trp13Gly) that was inherited from his mother and a missense variant c.683C>G (p.Ser228Trp) that was inherited from his father. The p.Trp13Gly variant is present in the ExAC database with an allelic frequency of 0.003383 and is not predicted to be extremely deleterious (Table 1). However, its location in the mitochondrial localization signal of the protein gives it functional relevance. In a recent report by Musante et al, the p.Trp13Gly variant was found to significantly reduce the level of WARS2 protein in the mitochondrial fraction, indicating that this mutation leads to mislocalization.^9^ The p.Ser228Trp variant is not present in the ExAC database. Using wInterVar, p.Trp13Gly had an adjusted score of likely pathogenic (PS1, PM3, BP4) and p.Ser228Trp had an adjusted score of likely pathogenic (PS3, PM2, PP3).

**Table 1:** Clinical characteristics of patients with biallelic mutations in *WARS2*

Column 1	Family 2‐V:7	Patient 1	Proband 1
WARS2 mutation Allelle 1 [pathogenecity prediction]	c.37T>G (p.Trp13Gly)	c.938A>T (p.Lys313Met)	c.37T>G (p.Trp13Gly) [deleterious by SIFT, benign by polyphen, polymorphism by MutationTaster, CADD Phred score of 17.58]
WARS2 mutation Allelle 2	c.325delA (p.Ser109Alafs*159)	c.298_300delCTT (p.Leu100del)	c.683C>G (p.Ser228Trp) [deleterious by SIFT, probably damaging by polyphen, disease causing by MutationTaster, and has a CADD Phred score of 34]
Clinical presentation	Intellectual disability	Infantile onset leukoencephalopathy	Infantile‐onset Parkinsonism
Gender	Female	Male	Male
Age‐of‐onset	Childhood	Infantile	1‐year‐old
Present age	17 years	24 y/o (deceased)	17 y/o
Ethnic origin	Iranian	European	European
Consanguinity	Yes (first cousins)	No	No
Height	150 cm (at time of examination; −2SD)	Not available	50.2 cm (birth, 50th centile)
Weight	52 cm (at time of examination; −2SD)	2850 g (birth, 12th centile)	3302 g (birth, 25th centile)
Head circumference	52 cm (at time of examination; −2SD)	35 cm (at birth, 34th centile); 16 months, 3rd centile	Not known
Dysmorphism	Long philtrum	None	None
Speech	Impaired	Can vocalize sounds; no spoken word	Hypophonia
Psychomotor development	Delayed	Delayed	Delayed
Cognitive ability	Moderate (IQ of 46)	Profound intellectual disability (not formally tested)	Normal
Neurological examination	Muscular weakness and ataxia	Axial hypotonia, appendicular hypertonia, hyper reflexia, and tremor	Dystonia, tremors in extremities that are responsive to Levodopa treatment
Seizures	None	Recurrent seizures, poorly controlled	None
MRI	Not described	Delayed myelination (7 months), generalized volume loss with patchy white matter signal abnormalities	Minimal non‐specific atrophy for age
Others	Aggressive behavior; Athetosis	Skin biopsy negative for lipofuscin; Athetosis	Increased and swollen mitochondria seen in muscle EM
Reference	9	8	This paper

### 3.4 Analysis of dermal fibroblasts

**Figure 2:**
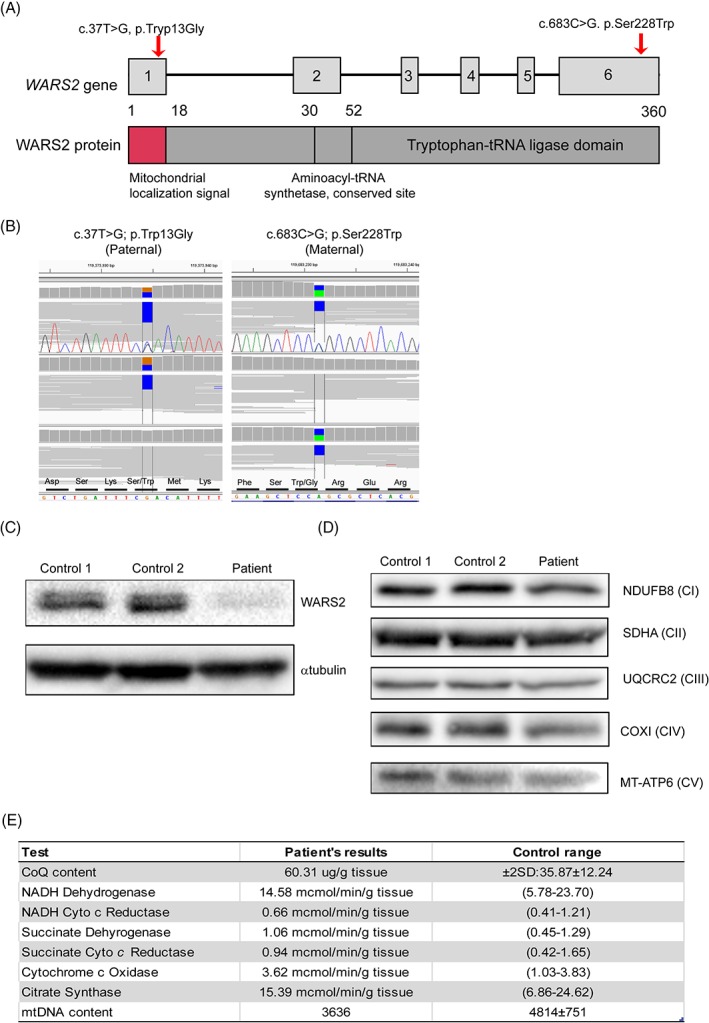
Molecular data and analysis of mitochondrial complex in dermal fibroblasts from patient. (A) Schematic diagram of *WARS2* gene and protein. (B) IGV images showing the mutations inherited from the patient's father and mother. Chromatograms from Sanger sequencing are superimposed. (C) Analysis of WARS2 expression in control and patient's dermal fibroblasts. (D) Steady state analysis of the respiratory chain subunits in 2 independent control fibroblast line compared to patient's fibroblasts: NDUFB8 (Complex I); SDHA (Complex II); UQCRRC2 (Complex III); COXI (Complex IV); and MT‐ATP6 (Complex V). (E) Values of tests done on muscle biopsy; normal values for control samples from age‐match controls are shown

Western blot analysis of patient fibroblasts revealed a marked decrease in WARS2 steady‐state protein levels relative to controls, demonstrating a functional consequence of the identified *WARS2* variants (Figure 2C). The steady state levels of OXPHOS subunits NDUFB8 (CI), UQCRC2 (CIII), COXI (CIV) and ATP6 (CV) were relatively unaffected in the patient as compared to controls (Figure 2C), using SDHA (CII) as a loading control since CII is entirely nuclear‐encoded.

### 3.5 Muscle biopsy analysis

A quadriceps muscle biopsy had non‐specific changes including mild variation in fiber size with rare atrophic fiber, and normal COX and SDH stains. Muscle electron microscopy demonstrated well preserved z‐bands and fibrils with minimally increased subsarcolemmal mitochondria, many which were mitochondria and some that vary in shape without paracrystalline inclusions. Muscle CoQ10 levels were 168% of control (Figure 1E, ±above the mean), consistent with mitochondrial proliferation. Measurement of individual enzyme activities of the mitochondrial respiratory chain (RC) enzymes revealed normal results (Figure 1E). Mitochondrial DNA content by quantitative polymerase chain reaction (qPCR) analysis was 76% of age‐ and tissue‐matched control.

## 4 DISCUSSION

Numerous publications have documented significant phenotypic variability caused by defects in mtARS genes.^6^, ^7^ Patients harboring biallelic mutation in *AARS2*, for example, may present with intrauterine or perinatal cardiomyopathy,^20^ adolescent onset ovario leukodystrophy^21^ or late‐onset neurodegeneration in patients with hereditary diffuse leukoencephalopathy with spheroids (HDLS).^22^ The explanation for the broad range of manifestations is unclear.

The latest mtARS to be linked to a disease is *WARS2*, based upon 2 independent case reports. Musante et al^9^ described 2 siblings with moderate delayed psychomotor development, speech impairment, ataxia, and athetoid movements. Theisen et al^8^ reported an individual harboring different biallelic missense mutations in *WARS2* who had developmental delay, infantile‐onset leukoencephalopathy, spasticity, hypotonia, tremor, muscle atrophy, and seizures.

Here, we present a fourth patient with compound heterozygous mutations in *WARS2* and a clinical presentation of infantile‐onset, Levodopa‐responsive Parkinsonism. While the clinical presentations vary substantially among the reported *WARS2* cases and our own patient, there are some shared similarities. The first family carried one of the alleles found in our patient (p.Trp13Gly), along with a frameshift mutation predicted to result in loss of function; the latter may account for the more severe intellectual disability seen in the affected siblings. MRI information was not provided to establish the presence or absence of leukoencephalopathy. The MRI of the patient reported by Theisen et al revealed a periventricular multifocal leukoencephalopathy, but the clinical presentation might have been compounded by perinatal insult.^8^ Both previous reports indicate intellectual disability, various neurological deficits, and symptoms including motor control disorders (ataxia, tremor, spasticity and athetoid movement). Table 1 compares the clinical characteristics of patients with biallelic *WARS2* mutations to date.

Theisen et al^8^ found impaired RC subunit expression in their patient with *WARS2* mutations, while we found no significant change in RC subunit expression or function in our patient's muscle tissue. This difference likely reflects the unique impact that a specific variant in *WARS2* can have on the resulting cellular and clinical phenotype, or different methodologies. In general, it is not uncommon for mtARS defects to have no effect on RC subunit expression in patient fibroblasts.^23^ Interestingly, the mtDNA content of the muscle is still lower than age‐matched control; this, together with the finding of increased mitochondrial CoQ10 content may suggest some compensation for the mtDNA depletion.

WES and whole genome SNP array data analysis for known variants and copy number variations associated with PD did not yield significant findings. The association of the patient's *WARS2* variants to Parkinsonism or PD may be further clarified by systematically interrogating available cohorts for such mutations. MRI imaging at the time of our evaluation revealed a structurally normal study without evidence of abnormal mineral or metal deposition in the deep brain nuclei or leukoencephalopathy, but rather minimal non‐specific atrophy. The temporal pattern of our patient's clinical progression has followed the prototypical course of adult idiopathic PD, except that it began 6 decades earlier. Specifically, our patient exhibited the typical early “honeymoon” response to Levodopa supplementation allowing for several years of excellent clinical response, followed by increasingly severe motor fluctuation associated with progressive nigrostriatal denervation that eventually led to the need for DBS at age 10 to achieve control of motor complications.

In conclusion, we expand the phenotypic spectrum of disorders caused by biallelic mutations in *WARS2* gene to include infantile‐onset, Levodopa‐responsive Parkinsonism and implicate *WARS2* dysfunction as a potential driver of mitochondrial dysfunction leading to nigrostriatal degeneration and Parkinsonism.

## Supporting information


**Appendix S1.** Materials and methods.

**Video S1.**

**Video S2.**

**Video S3.**

**Video S4.**

**Video S5.**

**Video S6.**

**Video S7.**
