# Supplementary material for: Biallelic mutations in mitochondrial tryptophanyl‐tRNA synthetase cause Levodopa‐responsive infantile‐onset Parkinsonism
Source: Clin Genet. 2018 Feb 5;93(3):712–8. doi: 10.1111/cge.13172 (PMC5828974; doi:10.1111/cge.13172)
Supplement: Supplementary file 1 — Appendix S1. Materials and methods. [file CGE-93-712-s001.docx]

Biallelic Mutations in Mitochondrial Tryptophanyl-tRNA Synthetase Cause Infantile-Onset Parkinsonism

Elizabeth A. Burke^1^, Steven J. Frucht^2^, Kyle Thompson^3^, Lynne A. Wolfe^1,5^, Tadafumi Yokoyama^4^, Maria Bertoni^1^, Yan Huang^5^, Murat Sincan^1^, David R. Adams^1,5^, Robert W. Taylor^3^, William A. Gahl^1,4,5^, Camilo Toro^1,5^, May Christine V. Malicdan^1,5^.

1 NIH Undiagnosed Diseases Program, Common Fund, Office of the Director, NIH and National Human Genome Research Institute, NIH, Bethesda, Maryland, USA

2 Movement Disorders Division, New York University Langone School of Medicine, New York, USA

3 Wellcome Centre for Mitochondrial Research, Institute of Neuroscience, The Medical School, Newcastle University, Newcastle upon Tyne, UK

4 Section of Human Biochemical Genetics, Medical Genetics Branch, National Human Genome Research Institute, NIH, Bethesda, Maryland, USA

5 Office of the Clinical Director, National Human Genome Research Institute, NIH, Bethesda, Maryland, USA

**Supplemental Data**

**Supplemental Methods**

***Clinical data and samples***

The patient, following informed and written consent, under the protocol 76-HG-0238, “Diagnosis and Treatment of Patients with Inborn Errors of Metabolism or Other Genetic Disorders” of the NIH-UDP Clinical data and patient samples were obtained in accordance with the ethical standards approved by the National Human Genome Research Institute (NHGRI) Institutional Review Board (IRB).

***Genetic Analysis***

High density whole genome SNP array methods were done as described (6).

Whole exome sequencing of both parents and the patient was performed at NIH Intramural Sequencing Center (NISC). SureSelectV5 (Agilent Technologies) was used for exome capture and subsequent sequencing was done on a HiSeq2000 instrument (Illumina Inc.) Sample library preparation, sequencing, and analysis were performed using the standard NIH Intramural Sequencing Center (NISC) pipeline (1) and Axeq Technologies (Seoul, South Korea). The analysis of whole exome sequencing data is described in Supplemental Methods.

Whole-exome sequence analysis was performed on genomic DNA that was isolated from the patient and his unaffected mother and father. The quality of whole exome data is shown in Supplemental table 1.

The sequencing reads then were filtered for quality, and aligned to human reference genome NCBI build 37 (hg19) using in-house developed pipelines, one based on Novoalign (Novocraft Technologies), and separately a diploid aligner (2) run on a commercial platform (Appistry Inc.). Variants were called with HaplotypeCaller and GenotypeGVCFs (2-4)). Annotations utilized snpEff (5) and a combination of publically available data sources (ExAC, GnomAD, ESP, 1000Genomes) and internal cohort statistics were utilized for variant filtration. We assessed the variants listed in the Variant Call Files (VCFs), which were filtered based on rarity (MAF <0.02, 95% confidence interval and homozygote count ≤ 25; UDP founders cohort population frequency data with variant allele count<8) (6), Mendelian segregation, and predicted deleteriousness, and prioritized based on coding effect (nonsynonymous, frameshift, stopgain, stoploss, startloss, inframe), proximity to splice sites (within 20 base pairs of a canonical splice site into the intron, or 5 base pairs into the exon), CADD v1.3 Phred scores (7) and Exomiser (8). The quality of alignment and genotype call of variants were checked using the Integrative Genome Viewer (<https://www.broadinstitute.org/igv/home)>. The final candidate variants were validated through Sanger sequencing. The primers GCAACGTCACTACGCTCTGA (fwd) and GGGATCCAGGGAAAAACACT (rev) were used to amplify the region of genomic DNA around the c.37T>G variant and the primers TCTCAATCACAGCATCTGCC (fwd) and AAAACAGAATGGTTGTGTGGG (rev) were used to amplify the region surrounding the c.683C>G variant in *WARS2* (NM_015836.3). Sanger dideoxy sequencing of the PCR products was executed by Macrogen. The resulting sequences were aligned using Sequencher v.5.0.1 (Gene Codes).

Variant pathogenicity was evaluated by SIFT, Polyphen, MutationTaster, CADD Phred, and wInterVar

***Analysis of Dermal Fibroblasts***

A culture of dermal fibroblasts from the patient was established as previously described (9). Control fibroblasts were purchased from Coriell Institute for Medical Research.

For quantification of WARS2 and oxidative phosphorylation in dermal fibroblasts, human fibroblasts were trypsinized, pelleted, and resuspended in cell lysis buffer as previously described (10), using the following antibodies: COXI (Abcam cat# ab14705), SDHA (Abcam cat# ab14715), UQCRC2 (Abcam cat# ab14745), NDUFB8 (Abcam cat# ab110242), MT-ATP6 (ProteinTech cat# 55313-1-AP), WARS2 (kind gift from Prof. Roger D. Cox, and from SAB1406979, Sigma Aldrich) and α-tubulin (Abcam cat# ab7291), and HRP-conjugated secondary antibodies (Dako Cytomation).

***Analysis of Skeletal Muscle Biopsy***

A piece of muscle was surgically obtained for enzymology studies (flash-frozen), histology (OCT-embedded), and electron microscopy (fixed in glutaraldehyde solution). Coenzyme Q10 (CoQ10) content was measured by high performance liquid chromatography with CoQ9 as an internal standard, and individual enzyme activities of the mitochondrial respiratory chain complex enzymes were measured by a diagnostic laboratory (Robert Guthrie Biochemical Genetics Laboratory, Buffalo, NY). Mitochondrial and nuclear DNA copy numbers were performed by a clinical lab using real-time quantitative (qPCR) specific primers for the mitochondrial tRNA^Leu(UUR)^ (*MT-TL1*) gene and the nuclear β-2-micoglobulin (*β2M*) gene (Baylor Medical Genetics Laboratory).

**Supplemental table 1.**

| SAMPLE | UDP1166 (proband) | UDP2553 (father) | UDP2552 (mother) |
| --- | --- | --- | --- |
| COVERAGE | 91.8 % | 90.8 % | 91.6 % |
| CALLS | 59587484 | 53314997 | 57036729 |
| HETEROZYGOSITY | 0.00067 | 0.00066 | 0.00067 |
| HET3 | 0.00098 | 0.00096 | 0.00096 |
| TI-TV RATIO | 2.283037395 | 2.299597039 | 2.325184863 |
| HET_HOMNR RATIO | 2.174044974 | 2.238255674 | 2.282106394 |
| chrM_ALIGNED | 4926 | 4466 | 4281 |
| TOTAL VARIANTS | 24187 | 23666 | 24298 |
| #VARIANTS IN DBSNP | 21026 | 20616 | 21143 |
| % VARIANTS IN DBSNP | 86.9% | 87.1% | 87.0% |
| TOTAL_READS | 125793648 | 96647825 | 102702181 |
| PF_READS | 125793648 | 96647825 | 102702181 |
| PCT_PF_READS | 100% | 100% | 100% |
| PF_READS_ALIGNED | 104370579 | 77115606 | 85352096 |
| PCT_PF_READS_ALIGNED | 0.829697 | 0.797903 | 0.831064 |
| PF_ALIGNED_BASES | 7497991742 | 5536843240 | 6191043273 |
| PF_HQ_ALIGNED_READS | 104045970 | 76850233 | 85094520 |
| PF_HQ_ALIGNED_BASES | 7477006237 | 5519775859 | 6174026693 |
| PF_HQ_ALIGNED_Q20_BASES | 7392669411 | 5460004807 | 6125239063 |
| PF_MISMATCH_RATE | 0.001229 | 0.00141 | 0.001234 |
| PF_HQ_ERROR_RATE | 0.001191 | 0.00137 | 0.001202 |
| PF_INDEL_RATE | 0.000063 | 0.000062 | 0.000064 |
| MEAN_READ_LENGTH | 72.754165 | 73.033177 | 73.684089 |
| READS_ALIGNED_IN_PAIRS | 102996604 | 76055099 | 84487613 |
| PCT_READS_ALIGNED_IN_PAIRS | 0.986836 | 0.986248 | 0.989872 |
| STRAND_BALANCE | 0.497705 | 0.494231 | 0.498163 |
| PCT_CHIMERAS | 0.002324 | 0.002039 | 0.004623 |
| SITES COVERED | 36950616 | 36975930 | 36974381 |
| DEPTH OF COVG | 121.7 | 89.5 | 99.9 |

**Supplemental table 2. List of other genes identified through whole exome sequencing**

| Gene | Inheritance | Variant | Comments |
| --- | --- | --- | --- |
| *SETD6 (*NM_001160305) | Homozygous recessive | p.Met288Ilefs*3 | Seen in ExAc (0.129%) |
| *CUL4A* (NM_001008895) | Compound heterozygous | c.665C>G; p.Ser222Cys  c.1004C>T; p.Ala335Val | Sp.Ala335Val seen in ExAc (0.102%) |
| *FRY* (NM_023037) | *De novo* | c.2202C>G; p.His734Gln | Seen in ExAc (0.001%) |
| *LRCH2* (NM_020871) | X-linked recessive | p.Gln382Pro | Seen in ExAc (0.005%) |
| *KDM5C* (NM_004187.3) | X-linked recessive | c.2368+16del; p? | Intronic.  Associated with X-linked Claes-Jensen type syndromic mental retardation |

**Supplemental video:**

Video segments recorded from age 2 to age 12 are shown serially in segments Suppl_1 thru Suppl_7. The first video was taken after levodopa had been started. DBS implantation occurred between Suppl_6 and Suppl_7. Residual resting tremor and dystonic

posturing of the arms and legs is evident. Video segments demonstrate impairment in gross and fine motor control, impaired ambulation and balance, as well as dysarthric and hypophonic speech. Time interval from the last L-Dopa dose varies across different video segments.

**URLs**

ExAc, <http://exac.broadinstitute.org>, accessed October 17, 2017

Mutation Taster, http://www.mutationtaster.org

PolyPhen, <http://genetics.bwh.harvard.edu/pph2/>

SIFT, <http://sift.jcvi.org>

wInterVar, <http://wintervar.wglab.org>

**References**

1. Program NCS. NIH Intramural Sequencing Center. Rockville, MD, 2014.

2. McKenna A, Hanna M, Banks E et al. The Genome Analysis Toolkit: a MapReduce framework for analyzing next-generation DNA sequencing data. Genome research 2010: 20: 1297-1303.

3. DePristo MA, Banks E, Poplin R et al. A framework for variation discovery and genotyping using next-generation DNA sequencing data. Nature genetics 2011: 43: 491-498.

4. Van der Auwera GA, Carneiro MO, Hartl C et al. From FastQ data to high confidence variant calls: the Genome Analysis Toolkit best practices pipeline. Current protocols in bioinformatics / editoral board, Andreas D Baxevanis [et al] 2013: 11: 11 10 11-11 10 33.

5. Cingolani P, Platts A, Wang le L et al. A program for annotating and predicting the effects of single nucleotide polymorphisms, SnpEff: SNPs in the genome of Drosophila melanogaster strain w1118; iso-2; iso-3. Fly 2012: 6: 80-92.

6. Gahl WA, Markello TC, Toro C et al. The National Institutes of Health Undiagnosed Diseases Program: insights into rare diseases. Genet Med 2012: 14: 51-59.

7. Kircher M, Witten DM, Jain P et al. A general framework for estimating the relative pathogenicity of human genetic variants. Nat Genet 2014: 46: 310-315.

8. Smedley D, Jacobsen JO, Jager M et al. Next-generation diagnostics and disease-gene discovery with the Exomiser. Nat Protoc 2015: 10: 2004-2015.

9. Malicdan MC, Vilboux T, Stephen J et al. Mutations in human homologue of chicken talpid3 gene (KIAA0586) cause a hybrid ciliopathy with overlapping features of Jeune and Joubert syndromes. J Med Genet 2015: 52: 830-839.

10. Thompson K, Majd H, Dallabona C et al. Recurrent De Novo Dominant Mutations in SLC25A4 Cause Severe Early-Onset Mitochondrial Disease and Loss of Mitochondrial DNA Copy Number. Am J Hum Genet 2016: 99: 860-876.
